# Why Using Molecularly Imprinted Polymers in Connection to Biosensors?

**DOI:** 10.3390/s17020246

**Published:** 2017-01-27

**Authors:** Bo Mattiasson, Gizem Ertürk

**Affiliations:** 1 CapSenze Biosystems AB, Scheelevägen 22, 22363 Lund, Sweden; ge@capsenze.se; 2 Department of Biotechnology, Lund University, Box 117, 221 00 Lund, Sweden

The area of biosensor-oriented research has grown rapidly during recent years. From a start with enzyme-based sensors, it soon moved over to affinity-based sensors, e.g., immuno-based sensors. As long as the analytical device was used for a single measurement, i.e., as a disposable, stability problems were connected to storage stability, but not to operational stability. However, when repeated assays were to be used, or even sensors for on-line monitoring, then operational stability also came into play [1].

To secure high operational stability, one often operates in enzyme technology studies with a surplus of enzymes. That means that the process is diffusion-controlled, and when enzyme molecules denature and lose activity, then earlier resting enzymes come into action [2].

For immuno-based assays, one can approach the stability problems in two ways. The initial studies were made with competitive binding assays using labeled antigens mixed with free antigens. Then, the number of antibody binding sites was limiting, and denaturation then resulted in decreased sensitivity of the assay. One can compensate for these negative effects by measuring the binding of a standardized samples of labeled antigen [3]. If this is taken as 100% signal, then assay of the native antigen in the competitive assay will stay constant, as seen in Figure 1. For direct binding assays, an excess of binding sites is needed, and denaturation of a fraction thus does not influence sensitivity since earlier resting antibodies come into play instead of those that denatured.

Most binding assays involving MIPs are at present direct binding assays; thus, one shall use a surplus of the binding sites.

There are many reasons why a sensor with an immobilized biomolecule (enzyme, antibody, etc.) is losing activity. Denaturation due to temperature effects is often regarded as a causative; therefore, a refrigerator is recommended for storage of the bioactive components. The low temperature tolerance restricts the use of enzyme-based sensors in, e.g., fermentation control. It has so far, in essence, been impossible to operate with enzyme-based biosensors directly in fermentation broth. Off-line analysis has been the alternative choice. Thermostable enzymes from extremophilic microorganisms may help to some extent, but not enough for standing heat sterilization.

In areas where an unbroken cold-chain cannot be guaranteed, then biosensors based on selectivity of biological macromolecules constitute a problem. That may be a problem in developing countries, esp. when analysis is planned in remote areas.

However, there are other problems that might appear, e.g., the presence of proteases in the medium to be analyzed (that was what happened in the experiment illustrated in Figure 1). Such enzymes may destroy the bioactive component on the sensor surface, thereby reducing the sensitivity and definitely destroying the reproducibility. There are examples when infections of microorganisms have led to the presence of proteases that quickly destroyed the sensor device (Figure 1).

One more factor that is causing problems is the presence of heavy metal ions. It has been documented that even when using *pro. analysis* quality of buffer chemicals used in enzyme technology processes when the enzymes are exposed to buffer for extended periods of time, the enzymes lose the activity as a result of reaction with heavy metal ions present in the buffer used.

Means to avoid the problems of bio-macromolecules have involved the use of small molecules with selective binding properties for certain analytes. Thus, amino-phenylboronate, which selectively binds vicinal hydroxyl groups, has been used for designing sensors for, e.g., glucose. It has even been proven that one can heat-sterilize a sensor surface with immobilized amino-phenylboronate and maintain the response pattern to the analyte, as seen in Figure 2 [4]. There are of course also some limitations with regard to selectivity. Synthesis of such low-molecular-weight compounds with selectivity is cumbersome and therefore expensive.

Yet another alternative was presented by Nieland and Enfors [5]. They used the approach to add the enzymes to the space between the sensing part of an oxygen electrode and the protecting membrane covering the electrode surface. By that arrangement, the enzyme was excluded from being exposed to high temperatures, as seen in Figure 3.

## Molecularly Imprinted Polymers (MIPs)

Molecularly imprinted polymers are relatively easy to produce—at least compared to the efforts one needs to put in for producing enzymes or antibodies. Most enzymes used are of microbial origin, and very often much screening work is needed before a suitable enzyme is recognized. Cloning, expression, and production are most often utilized in order to produce large enough amounts of enzymes. With regard to antibodies, there are two possibilities: one is the immunization of animals and subsequent purification of the polyclonal antibodies. The other alternative is the production of monoclonal antibodies—a time consuming and usually expensive process.

When producing MIPs, a few criteria are of special interest to meet. Selectivity, binding strength (which is reflected in the sensitivity of the assay), the ability to regenerate the MIP after usage, and stability, both operational and storage.

In the by-now classical work on “plastibodies,” it was found that MIPs were fully comparable to regular antibodies with regard to sensitivity [6,7]. Similar results have been reported for microcontact imprinting of proteins where sensitivity and selectivity was even better than what was measured for the commercial antibodies [8,9].

Molecularly imprinted polymers are said to be stable and can stand treatment of extremes of pH, organic base, and autoclave treatment [10]. An interesting aspect of MIPs is that one can modify the polymer properties by changing monomers to build the polymer. When introducing monomers that are often used for making stimuli responsive polymers, it is possible to facilitate release of bound material by simply changing one environmental parameter, often temperature. Thus, when N-isopropylacrylamide (NIPAm) is one of the co-monomers building up the MIP, then temperature sensitive conformation is obtained. However, when temperature returns to the starting point, then the conformation also returns to what was relevant before binding took place.

When MIPs were first described, block polymerization was used with a subsequent crushing and fractionation [11]. The smaller the particles were, the larger the surface area that was exposed and that could be used in the binding assays. Later on, spherical microparticles were produced [12], and more recent development has been described for the production of imprinted nanoparticles, with sizes of, e.g., those of antibodies [7]. Moreover, surface imprinting offers an option that can be used in connection to biosensors [13,14,15]. All of these alternatives can be used when designing biosensors, and papers in this issue will illustrate several of these.

MIPs can be applied in sensor systems in different ways. Forming the MIPs directly on the electrode surface is a common method; alternatively, small MIP particles can be added and fixed to the electrode, either by forming a composite via polymerization or by covalent coupling [16]. A recent development is the design of nano-MIPs—structures of sizes comparable to that of antibodies [7]. When small MIPs are designed, the number of crosslinks may become important for the stability of the MIP [17]. 

Still another area of applications of MIPs in analysis is the use as solid phase extraction medium. After a proper period of capture of the target molecule, an enriched fraction can be washed off the MIP and subsequently analyzed.

Experiences from affinity separation using MIPs will certainly stimulate to broaden the analytical applications of MIP.

The present volume is focused around the combination of biosensors and MIPs. A range of different targets is analyzed, and several sensor configurations are presented. From the data presented, it looks as if MIPs will become important when manufacturing selective sensors in the future. MIP-based sensors are often classified as “biosensors,” even if the MIP is not actually a bio part.

## Figures and Tables

**Figure 1:**
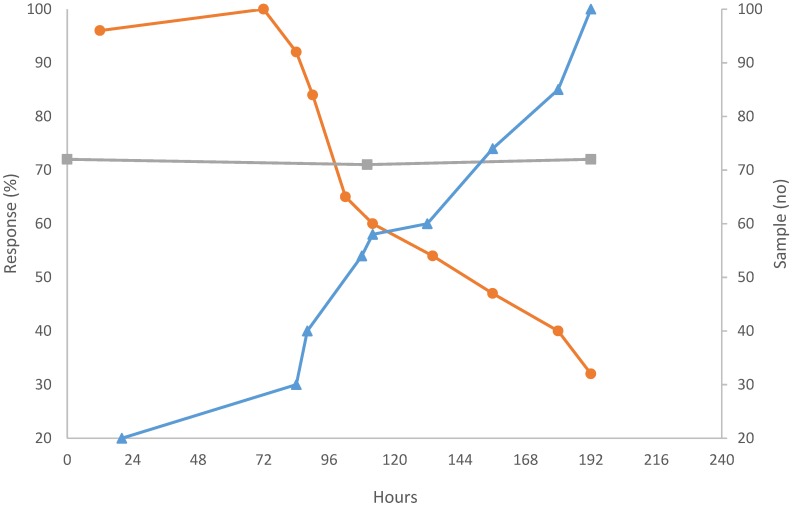
Response stability of antibody-Sepharose CL 4-B preparation. Orange line (circle) shows the peak height obtained when assaying a 100% sample. Blue line (triangle) shows number of assay cycles run. Grey line (square) shows the peak height in percent of a preceding pure aggregate pulse obtained for reference samples containing 40 µg HSA/mL sample (Modified from [3]).

**Figure 2:**
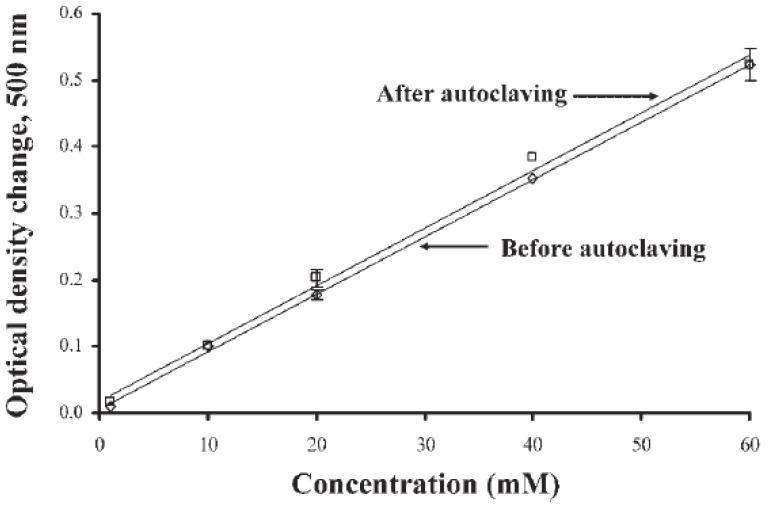
Sensitivity of N-acryloyl-m-aminophenylboronic acid–acrylamide copolymer (NAA PBA-Am copolymer) gel to glucose in 50 mM sodium phosphate buffer pH 7.3 before (◊) and after (□) autoclaving. Gel thickness is 350 nm. (Reproduced from [4] with permission).

**Figure 3:**
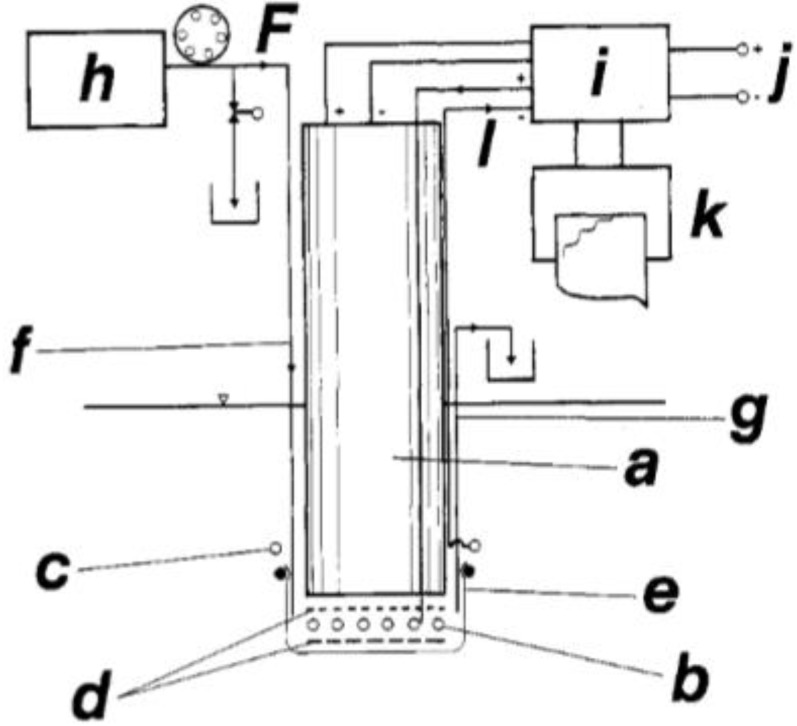
Main parts of the externally buffered enzyme electrode: a. oxygen electrode; b. Pt gauze with immobilized enzymes; c. Pt cathode; d. nylon nets; e. dialysis membrane; f. in-going buffer stream; g. buffer effluent; h. buffer reservoir; i. PID controller; j. reference potential; k. recorder; l. electrolysis current; F. buffer flow. (Reproduced from [5] with permission).
